# First report of V1016I, F1534C and V410L *kdr* mutations associated with pyrethroid resistance in *Aedes aegypti* populations from Niamey, Niger

**DOI:** 10.1371/journal.pone.0304550

**Published:** 2024-05-29

**Authors:** Abdoul-Aziz Maiga, Aboubacar Sombié, Nicolas Zanré, Félix Yaméogo, Souleymane Iro, Jean Testa, Antoine Sanon, Ousmane Koita, Hirotaka Kanuka, Philip J. McCall, David Weetman, Athanase Badolo

**Affiliations:** 1 Laboratoire d’Entomologie Fondamentale et Appliquée, Université Joseph Ki-Zerbo, Ouagadougou, Burkina Faso; 2 Unité de Parasitologie et d’Entomologie Médicale, Centre de Recherche Médicale et Sanitaire, Niamey, Niger; 3 Faculté de Médecine, Université Côte d’Azur, Côte d’Azur, France; 4 Laboratoire de Biologie Moléculaire Appliquée, Université des Sciences, des Techniques et Technologies de Bamako, Bamako, Mali; 5 Center for Medical Entomology, The Jikei University School of Medicine, Tokyo, Japan; 6 Department of Tropical Medicine, The Jikei University School of Medicine, Tokyo, Japan; 7 Department of Vector Biology, Liverpool School of Tropical Medicine, Liverpool, United Kingdom; United States Department of Agriculture, UNITED STATES

## Abstract

**Background:**

*Ae*. *aegypti* is the vector of important μ arboviruses, including dengue, Zika, chikungunya and yellow fever. Despite not being specifically targeted by insecticide-based control programs in West Africa, resistance to insecticides in *Ae*. *aegypti* has been reported in countries within this region. In this study, we investigated the status and mechanisms of *Ae*. *aegypti* resistance in Niamey, the capital of Niger. This research aims to provide baseline data necessary for arbovirus outbreak prevention and preparedness in the country.

**Methods:**

Ovitraps were used to collect *Ae*. *aegypti* eggs, which were subsequently hatched in the insectary for bioassay tests. The hatched larvae were then reared to 3–5-day-old adults for WHO tube and CDC bottle bioassays, including synergist tests. The kdr mutations F1534C, V1016I, and V410L were genotyped using allele-specific PCR and TaqMan qPCR methods.

**Results:**

*Ae*. *aegypti* from Niamey exhibited moderate resistance to pyrethroids but susceptibility to organophosphates and carbamates. The *kdr* mutations, F1534C, V1016I and V410L were detected with the resistant tri-locus haplotype 1534C+1016L+410L associated with both permethrin and deltamethrin resistance. Whereas the homozygote tri-locus resistant genotype 1534CC+1016LL+410LL was linked only to permethrin resistance. The involvement of oxidase and esterase enzymes in resistance mechanisms was suggested by partial restoration of mosquitoes’ susceptibility to pyrethroids in synergist bioassays.

**Conclusion:**

This study is the first report of *Ae*. *aegypti* resistance to pyrethroid insecticides in Niamey. The resistance is underpinned by target site mutations and potentially involves metabolic enzymes. The observed resistance to pyrethroids coupled with susceptibility to other insecticides, provides data to support evidence-based decision-making for *Ae*. *aegypti* control in Niger.

## Introduction

*Aedes aegypti* is a mosquito that spreads several viral diseases including dengue, zika, chikungunya and yellow fever. Among these, dengue and yellow fever are the most important arboviruses affecting West Africa [1]. In recent years, dengue outbreaks and sporadic cases have been reported in the region, notably in Burkina Faso [2], Côte d’Ivoire [3], Senegal [4] and Ghana [5]. Yellow fever epidemics or cases have also occurred in Ghana [6], Nigeria [7] and Côte d’Ivoire [8].

With vaccines lacking, of limited efficacy or in limited supply, the control of these diseases relies on vector control using insecticidal interventions against immature and adult stages in combination with larval source reduction [9,10]. Understanding the resting behaviour (endophily or exophily), blood-feeding patterns and the preferential breeding habitats of immature stages as well as the insecticide resistance status are keys for effective vector control implementation and outbreaks preparedness [11–13]. West Africa is a region where dengue cases are predicted to increase in the coming years [14]. Despite recent reports on *Ae*. *aegypti* insecticide resistance and bionomics [15], data are still needed in most countries of West Africa to support vector control [16].

Resistance to insecticides is commonly mediated by target site mutations and metabolic enzymes. Target site ‘‘knock down resistance” (*kdr*) mutations result in a structural modification of the gene encoding the Voltage Gated Sodium Channel (*Vgsc*) that is targeted by pyrethroid insecticides and DDT [17]. More than 12 *kdr* mutations have been reported in the *Ae*. *aegypti* VGSC worldwide [18,19]. In West Africa, two *kdr* mutations, V1016I and F1534C have been reported in Cape Verde [20] whereas in Nigeria, S989P and F1534C kdr mutations are reported [21]. Three *kdr* mutations, V410L, V1016I and F1534C have been reported from Burkina Faso [22,23], Côte d’Ivoire [24] and Ghana [25].

Although dengue is not considered endemic in Niger, a first documented imported case during august of 2022 in Niamey [26] should serve as a warning. This is particularly important because neighbouring countries with similar ecological conditions are experiencing dengue outbreaks and regular dengue cases [1]. Vector control remains limited to nationwide regular distribution of long lasting bednets as part of Malaria control strategy [27]. Niger needs to be prepared and to reinforce capacities for early case diagnosis and vector control. This study aimed to document insecticide susceptibility of *Ae*. *aegypti* from Niamey against common insecticides and investigate the mechanisms which may be involved in resistance, to begin to fill the gap of data on the insecticide resistance in Niger.

## Materials and methods

### Sampling sites

*Ae*. *aegypti* eggs were collected using ovitraps from 5 sites located in Niamey, the capital city of Niger, from August to October of 2019, during the rainy season. Niamey is located in the south-western part of the country, has an area of 255 km^2^ with 1,565,056 inhabitants [28]. The climate is of Sahelian type with a rainy season lasting from June to October and an annual rainfall average of 540 mm. Niamey city is composed of five municipalities or communes and the collections were made at Gamkalé, Kombo, Talladié, INJS and Banifandou 2, situated within three of the municipalities. Gamkalé (13°29’28.5”N, 002°07’15.4”E) and Kombo (13°30’45.4”N, 002°05’51.2”E) are situated in Commune IV of Niamey on the left bank of the Niger River. Talladjé (13°29’46.8”N, 002°09’34.0”E) is also situated in Commune IV but not near the river. INJS (13°30’27.8”N, 002°05’50.8”E) is located in Commune V on the right bank of the river and Banifandou 2 (13°32’20.8”N, 002°08’17.3”E) is situated in Commune II. Gamkalé, Kombo and INJS are characterized by vegetable cropping alongside the river with use of pesticides. By contrast, Talladjé and Banifandou 2 are characterized by high human density and are more urbanized with pesticide use primarily via household insecticides for personal protection against mosquito bites.

#### WHO bioassays and CDC bottles tests with synergists

In total, 106 ovitraps were placed in the gardens or household yards of the 5 sites, after oral informed consent was obtained from the owners of the gardens at Gamkalé & INJS, and from the owners of houses in the remaining three sites.

The ovitraps were made of plastic containers in which filter paper was placed for egg-laying and water from two-day old mango leaf infusion was added. Ovitraps were collected three days later, and filter papers with eggs were dried at room temperature for twenty-four hours, placed in a sealed plastic bag and transported to the insectary of Université Joseph KI-ZERBO, Burkina Faso, for hatching and bioassays. Eggs were pooled across the 5 sites of collection and were hatched in a container with distilled water under controlled insectary conditions of 27.7 ± 1.4° C temperature, 79.1 ± 5.5% of relative humidity and a 12: 12 (light: dark) photoperiod. Hatched larvae were reared to adults using Tetramin® and emerged adults were provided with 10% sugar solution until 3 to 5 days old mosquitoes were obtained for bioassay tests.

Due to the absence of WHO-recommended doses for *Ae*. *aegypti* mosquitoes at the time of bioassays, *Anopheles* diagnostic doses were used [29]. Though these doses are slightly higher than *Aedes* doses and so provide a conservative assessment of resistance, they have been commonly used [13,18,25,30]. Insecticide impregnated papers including permethrin 0.75%, deltamethrin 0.05%, Malathion 5%, Pirimiphos-Methyl 0.25% and Bendiocarb 0.1% ordered from the WHO reference Center in Malaysia, were tested against *Ae*. *aegypti* populations. For each insecticide test, four tubes of 20–25 female mosquitoes of 3 to 5 days old and two control tubes were used. After one hour exposure to insecticide, mosquitoes were removed from exposure tubes to observation tubes and kept for 24 hours. The number of mosquitoes dead after 24 hours was counted and the mortality calculated and corrected with Abbott’s formula [31] when mortality in the control was between 5 to 20%. Survivors and dead mosquitoes from bioassays were stored in 1.5 ml tubes over silica gel and kept at– 20°C for molecular analysis. Bioassays were performed under the same temperature and humidity conditions as mosquito rearing (27.7 ± 1.4° C temperature, 79.1 ± 5.5% of relative humidity).

Bottle bioassays with and without synergists were also performed to assess the effect of synergist pre-exposure on pyrethroid insecticide efficacy and to assess the possible involvement of metabolic enzymes in pyrethroid resistance. Susceptibility tests were performed following the CDC bottle bioassay guidelines [32], but with modifications according to the WHO guidelines. [33] Insecticide stock solutions were prepared using acetone as solvent, at concentrations of 15 μg/ml for permethrin and 10 μg/ml for deltamethrin. Synergist stock solutions were prepared at concentrations of 400 μg/ml for PBO (Piperonyl butoxide) and 125 μg/ml for DEF (S.S.S-tributylphosphorotrithioate). From each insecticide and synergist solution, 1 ml per bottle was used to coat 4 bottles of 250 ml for the insecticide and 2 control bottles coated with acetone only. Coated bottles were dried at room temperature for 24 hours and kept in the fridge until use. For each bioassay using synergist, 100–125 non-blood-fed female mosquitoes of 3–5 days old were first exposed to synergists (synergist exposure bottles) at a rate of 25 mosquitoes per bottle for 1 hour, then transferred to holding cups before transfer to 4 replicate pyrethroid-coated bottles and one acetone-coated bottle as a negative control for 1 hour exposure [32,33]. In parallel, direct insecticide exposure bioassay tests were done with 100–125 mosquitoes first exposed to acetone (as synergist control bottles) for 1 hour, then transferred to holding cups before transfer to 4 replicate pyrethroid insecticides coated bottles for 1 hour and one acetone coated bottle as negative control. After exposure, the mortality was recorded 24h after, and Abott’s correction was made if the mortality rate in the control was between 3 and 10%. Dead and alive mosquitoes were kept in 1.5 ml tube over silica gel and stored at– 20°C. Bottle bioassays tests were done under the same controlled insectary conditions as the tube bioassays (27.7 ± 1.4° C temperature, 79.1 ± 5.5% of relative humidity).

### DNA extraction

Each mosquito was homogenised in 100 μl of buffer A (containing 0.1M tris pH 9.0, 0.1 M EDTA, 1% Sodium DodécylSulfate (SDS) and 0.5% Diethyl Pyrocarbonate (DEPC) with a sterilized pellet mixer. The homogenate was incubated at 70° C for 30 min in a block incubator (Labnet Dry Bath, Dual block, Ref D1302), then 22.4 μl of 5M of potassium acetate (KoAc) was added. The mixture was vortexed and cooled on ice for 30 min. The mixture was centrifuged for 15.000 rpm at 4° C for 15 min, after which 90 μl of supernatant was collected and 45 μl of isopropanol added. After vortexing, the mixture was centrifuged at 15,000 rpm at 4° C for 20 min. After centrifugation, the supernatant was discarded, and the pellet was rinsed with 200 μl of 70% ethanol. This mixture was centrifuged for 15.000 rpm at 4° C for 5 min, the supernatant discarded. The pellet was dried before being dissolved in 50 μl of Tris-EDTA pH 8.0 buffer.

### Allele-specific—PCR for the detection of F1534C, V1016I and V410L mutations

A total of 202 mosquitoes including alive and dead from permethrin 0.75% and deltamethrin 0.05% were chosen for genotyping the F1534C, V1016I and V410L *kdr* mutations by using AS-PCR methods. Allele Specific PCR procedures for the detection of V1016I and F1534C *kdr* mutations used the conditions detailed in Sombié et *al*. [34]. Detection of the V410L *kdr* mutation was performed following the protocol of Granada *et al*. [35]with modifications in the PCR conditions described in Sombié *et al* [22].

Two PCRs were used to genotyping the V and L alleles of the V410L *kdr* mutation using the primers listed in S1 Table. Each PCR was performed with a reactional volume of 12.5 including 1μl of Target DNA, 2.5 μl of primer’s mix (0.3 μM), 2.75 μl of sterile water and 6.25 μl of taq polymerase mix (AmpliTaq Gold®, Master mix, thermo Fischer Scientific). The PCR conditions were: initial denaturation for 7 minutes at 95°C, 35 cycles of extension at 95°C for 30s, 60°C for 30s and 72°C for 1-minute, terminal elongation at 72°C for 7 minutes.

### TaqMan detection of F1534C, V1016I and V410L *kdr* mutations

A total of 50 specimens of F0 mosquitoes (i.e., raised from field collected eggs), were screened using TaqMan qPCR to estimate the *kdr* mutations frequencies in the population. Reactions were performed in 96 well plates by adding 5 μl of TaqMan gene expression SensiMix (Applied Biosystem, Foster City, USA), 0.125 μl of primer/probe, 3.875 μl of molecular grade sterile water and 1 μl of the DNA extract [13]. Reactions were run in a separate reaction for each *kdr* locus on an Agilent MX3000P qPCR thermal cycler using cycling conditions of an initial denaturation of 10 min at 95° C, followed by 40 cycles of 92° C for 15 min and 60° C for 1 min.

### Statistical analysis

Mosquito susceptibility to insecticides in WHO tubes bioassay was interpreted according to WHO guidelines [29]. Mortality rates between 98–100% indicated susceptibility, mortality rates from 90 to 97% indicated possible resistance and mortality rates less than 90% indicated confirmed resistance. The mortalities of bottles bioassay with synergist were also interpreted according to WHO guidelines [33]: ≥98% mortality indicates full restoration of susceptibility; <98% but ≥90% increase in mortality indicates partial effect of synergist and enzyme involvement; < 90% mortality indicates no effect of synergist and no involvement of enzymes targeted. We used Generalized Linear Models (GLM) with binomial link function [36] using R package “stats version 4.0.5” to assess the effect of insecticide and their synergists on the mortality of *Ae*. *aegypti*. *Kdr* allele and genotype frequencies were calculated and Fisher’s exact two-sided test in R software version 4.0.5 was used to assess the association between *kdr* individual, tri-locus genotypes and mortality.

## Results

### *Ae*. *aegypti* susceptibility to insecticides in Niamey

**Fig 1:**
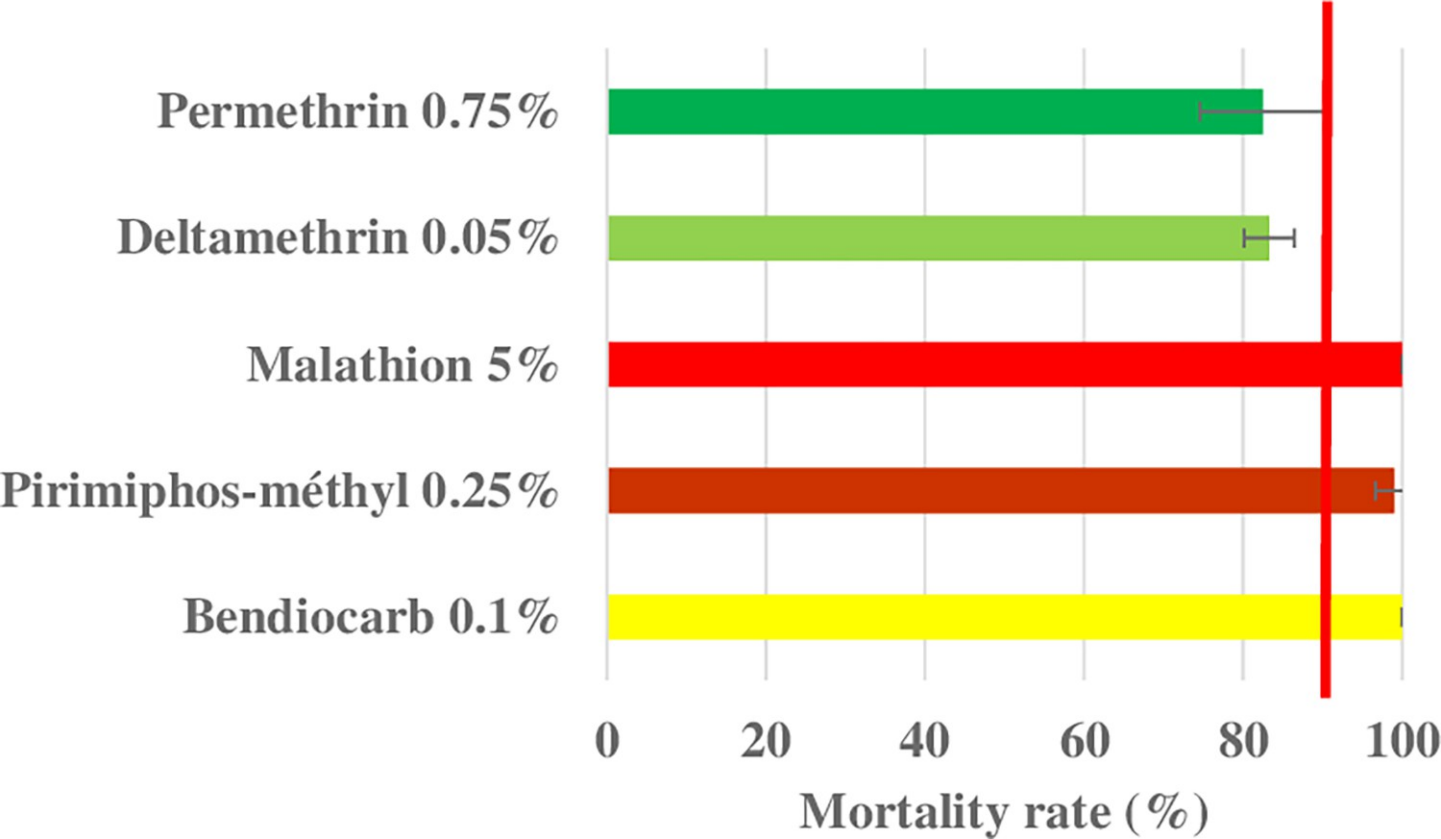
WHO tubes bioassay mortality of *Ae*. *aegypti* exposure to insecticides. The red line indicates the WHO resistance thresholds. Error bars represent 95% confidence interval of the mean.

WHO bioassays showed that *Ae*. *aegypti* mosquitoes from Niamey were susceptible to malathion and bendiocarb with 100% mortality rates, and with mortality rate of 99% to pirimiphos-methyl [CL:97.04–100]. Conversely, the mosquitoes were resistant to permethrin and deltamethrin with mortality rates of 82.52% [CL: 76.06–88.98] and 83.26% [CL: 80.64–85.8], respectively (Fig 1 and S2 Table).

### Alleles and genotypes of *kdr* mutations and their association with pyrethroid resistance

**Fig 2:**
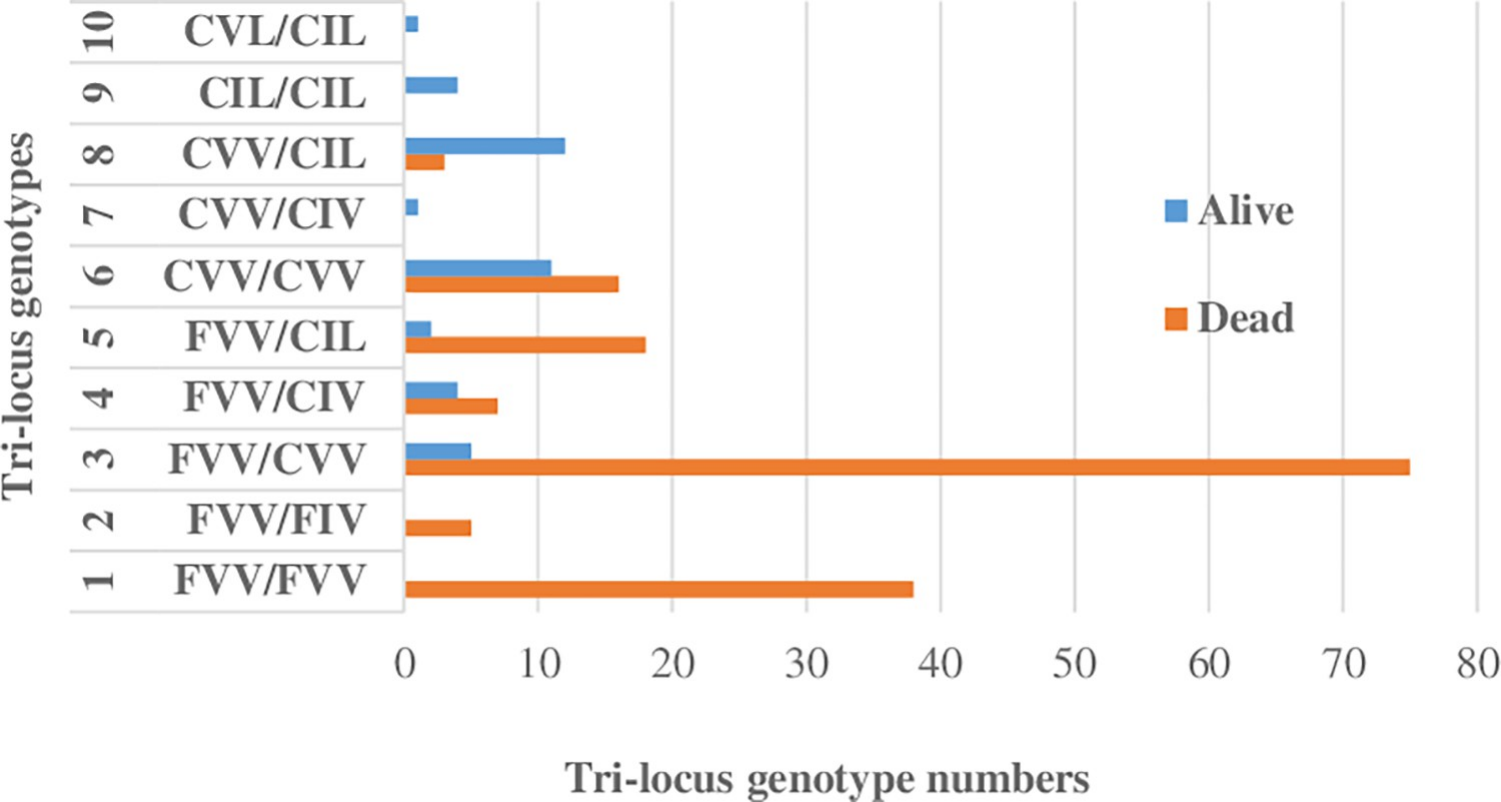
Number of tri-locus genotype combination of F1534C, V1016I and V410L *kdr* mutations in dead and alive pyrethroid-exposed *Ae*. *aegypti*.

**Fig 3:**
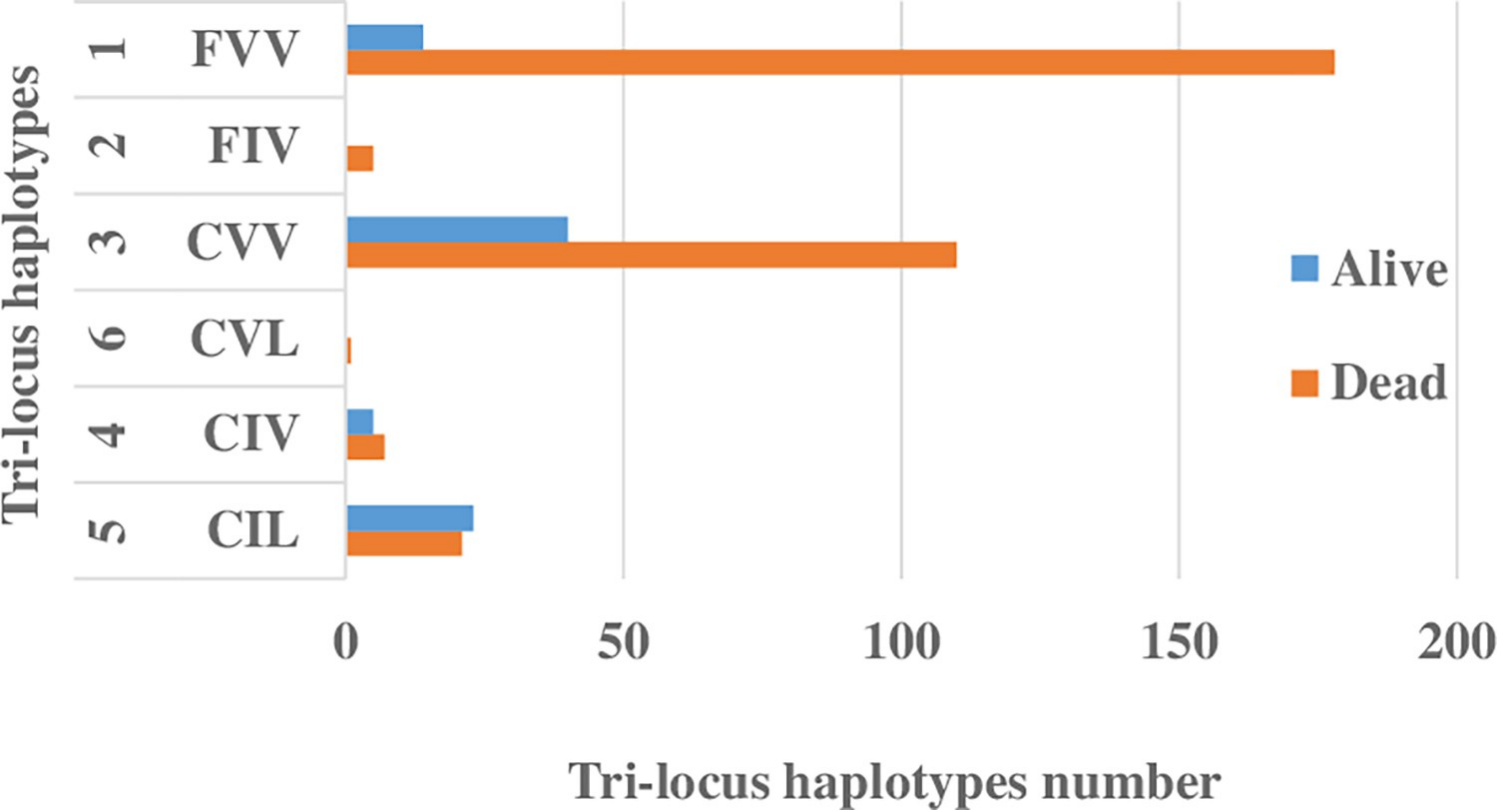
Number of tri-locus haplotype of F1534C, V1016I and V410L *kdr* mutations in dead and alive pyrethroid-exposed *Ae*. *aegypti*.

*Kdr* mutant allele frequencies were 0.30 for 1534C and 0.04 for both 1016I and 410Lin the cohort genotyped using TaqMan. Those in the cohort exposed to pyrethroids were significantly higher, but the same alleles were detected in each assay, and the frequency differences likely reflect inter-cohort differences (Tables 1 and S6). Ten genotypes were found out of 27 possible genotype combinations across the three *kdr* mutations F1534C, V1016I and V410L based on AS-PCR assay (Fig 2). The most common genotype was the single 1534 mutant FC/VV/VV at 39.6%, the triple-homozygote mutant for the three mutations CC/II/LL was found at around 2% while the triple homozygote wild-type FF/VV/VV was recorded at 18.8%. From these genotypes, six haplotypes were recorded with relative proportions of: FVV (47.5%), CVV (37.1%), CIL (10.9%), CIV (3%), FIV (1.2%) and CVL (0.2%) (Fig 3).

**Table 1:** Genotypes and, *kdr* allele frequencies of F1534C, V1016I and V410L mutations with 95% confidence intervals detected by TaqMan in F0 *Ae*. *aegypti* and by Allele Specific PCR in F1&F2 mosquitoes used in bioassays.

Method	Number of mosquitoes	F1534C genotype			Freq. of C allele	V1016I genotype			Freq. of I allele	V410L genotype			Freq. of L allele
		CC	FC	FF		II	VI	VV		LL	VL	VV	
**TaqMan**	50	8	14	28	0.3	00	4	46	0.04	00	4	46	0.04
**C.I.**				0.21–0.40				0.01–0.10				0.01–0.10		
**AS-PCR**	202	48	111	43	0.51	4	53	145	0.15	5	35	162	0.11
**C.I.**				0.46–0.56				0.12–0.19				0.08–0.15	

The tri-locus genotypes CC/VI/VL, CC/II/LL and FC/VI/VV were significantly associated with permethrin survival compared to the reference wild type genotype FF/VV/VV for which there were no survivors (Tables 2 and S3). The tri-locus haplotypes CIL, CVV and CIV were significantly associated with permethrin resistance with respectively p **< <**0.001, p = 0.014 and p = 0.016. (S4 and S3 Tables).

**Table 2:** Genotypes and their association with resistance to permethrin of *Aedes aegypti*.

Number	Genotypes	Phenotypes		Fisher exact P-value
		Dead (Susceptible)	Alive (Resistant)	
**1**	FF/VV/VV	16	0	Reference
**2**	FF/VI/VV	4	0	1
**3**	FC/VV/VV	35	2	0.570
**4**	**FC/VI/VV**	6	3	**0.037**
**5**	FC/VI/VL	10	0	1
**6**	CC/VV/VV	9	3	0.067
**7**	CC/VI/VV	0	1	0.059
**8**	**CC/VI/VL**	1	7	**<0.001**
**9**	**CC/II/LL**	0	4	**<0.001**
**10**	CC/VI/LL	0	0	**-**

Significantly associated genotypes are highlighted in bold.

The tri-locus genotypes CC/VV/VV, CC/VI/VL and CC/VI/LL showed significant association with deltamethrin resistance compared to the reference wild type genotype FF/VV/VV for which again there were no survivors (Tables 3 and S3). Unlike the permethrin dataset, the homozygote resistant genotype CC/II/LL was not present in any mosquitoes tested with deltamethrin. The tri-locus haplotypes CVV and CIL were significantly associated with deltamethrin resistance with respectively p **<<** 0.001 and p < 0.001 (S3 and S5 Tables).

**Table 3:** Genotypes and their association with resistance to deltamethrin of *Ae*. *aegypti*.

Number	Genotypes	Phenotypes		Fisher exact P-value
		Dead (Susceptible)	Alive (Resistant)	
**1**	FF/VV/VV	22	0	Reference
**2**	FF/VI/VV	1	0	1
**3**	FC/VV/VV	40	3	0.318
**4**	FC/VI/VV	1	1	0.083
**5**	FC/VI/VL	8	2	0.091
**6**	**CC/VV/VV**	7	8	**<0.001**
**7**	CC/VI/VV	0	0	**-**
**8**	**CC/VI/VL**	2	5	**<0.001**
**9**	CC/II/LL	0	0	**-**
**10**	**CC/VI/LL**	0	1	**0.043**

Significantly associated genotypes are highlighted in bold.

### Effect of synergists on pyrethroid efficacy

**Fig 4:**
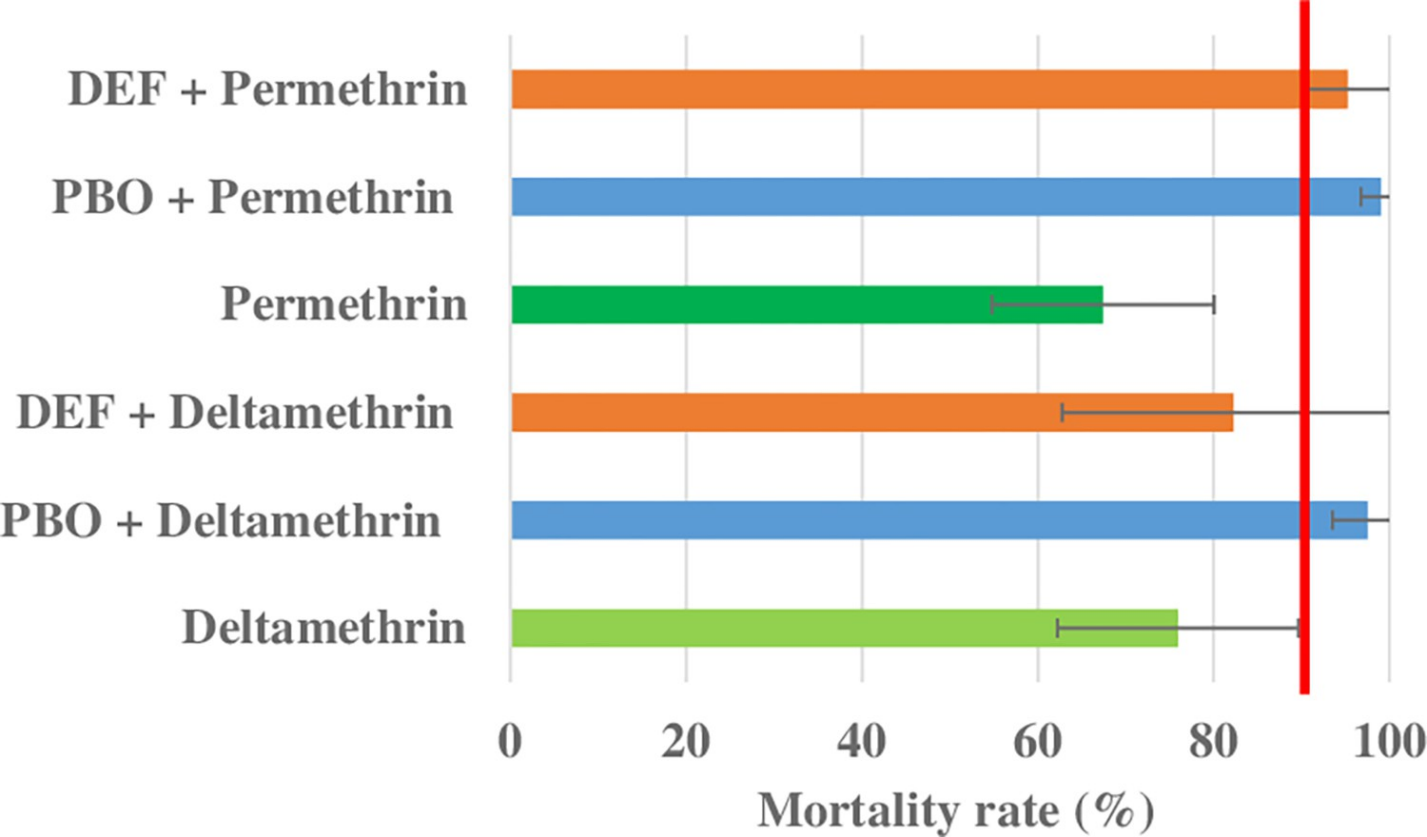
Bottle bioassay mortality of *Ae*. *aegypti* exposed to deltamethrin (10 μg/ml) and permethrin (15 μg/ml) insecticides with PBO and DEF synergists. The red line indicates the resistance thresholds. Error bars represent 95% confidence interval of the mean.

Pre-exposure to synergists increases mortality to permethrin and deltamethrin in *Ae*. *aegypti* from Niamey. Exposure to diagnostic concentrations of permethrin and deltamethrin resulted in 67.41% and 75.91% mortality, respectively (Fig 4 and S6 Table). Both PBO and DEF synergists significantly increased susceptibility to permethrin with respective mortality rates of 99.03% (p < 0.001) and 95.21% (p < 0.001) (Table 4 and Fig 4). Whilst PBO completely restored susceptibility to permethrin, DEF enhanced mortality rate but was still below the susceptibility threshold, suggesting a partial restoration of susceptibility. In contrast, only PBO significantly increased mosquito susceptibility with deltamethrin (mortality rate from 75.91% to 97.5%, p = 0.001) with a partial restoration of susceptibility, whereas DEF did not significantly increase mosquito susceptibility to deltamethrin with a mortality rate of 82.22% (p = 0.30) (Table 4 and Fig 4).

**Table 4:** Generalised linear model of the mortality of *Ae*. *aegypti* exposure to insecticides and synergists.

Predictors	Estimate	95% CL	z-value	Pr(>|z|)
**Intercept**	**0.736**	**[0.328–1.145]**	**3.531**	**<0.001**
**Insecticide [Permethrin]**				
**Permethrin + DEF**	**2.327**	**[1.342–3.313]**	**4.628**	**<0.001**
Permethrin + PBO	**3.859**	**[1.847–5.871]**	**3.759**	**<0.001**
**Intercept**	**1.173**	**[0.733–1.613]**	**5.225**	**<0.001**
**Insecticide [Deltamethrin]**				
**Deltamethrin + PBO**	**2.541**	**[1.071–4.011]**	**3.388**	**0.001**
**Deltamethrin + DEF**	0.356	[-0.317–1.029]	1.036	0.300

Reference factor levels of predictors are shown in brackets, with beta and effect size estimates, confidence intervals, z-value and associated probabilities for predictors included in the model. Significant predictor terms are shown in bold.

## Discussion

This study reports for the first time the resistance to pyrethroid insecticides in *Ae*. *aegypti* populations from Niamey, Niger. Three *kdr* mutations (F1534C, V1016I, and V410L) have been recorded, and oxidase and esterase detoxification enzymes (inferred from synergist assays) appear to be associated with the pyrethroid resistance phenotypes. The population was found to be susceptible to carbamate and organophosphate insecticides and these might represent useful options for insecticidal spray interventions in Niamey. Though the study is limited to Niamey, the presence of multiple insecticide resistance mechanisms in *Ae*. *aegypti* mosquito populations is a concern for vector control programs in this urban setting where a dengue outbreak is perhaps most likely to occur. A resistance surveillance program is needed here for *Ae*. *aegypti* control, to help toward preparedness for dengue prevention and outbreaks.

Though *Ae*. *aegypti* has not been a specific target of vector control, insecticide use in urban agriculture, and in domestic environments, may have played a role in resistance development. The localities of Gamkallé, Kombo and INJS are situated near the Niger River where vegetables are cultivated with pesticides and fertilizers used for crops protection. That may play some role in the development of insecticide resistance, but given the adaptation of *Ae*. *aegypti to* artificial breeding containers this is probably of limited importance [37]. There is no history of indoor residual spraying in Niger, and only insecticide treated bednets have been scaled up as malaria control strategy with bednet distribution every three years and the first largest distribution in 2006 [38]. Despite the exophilic behaviour of *Ae*. *aegypti* in West Africa [12,39,40], bednet exposure may also have played a role in the development of *Ae*. *aegypti* insecticide resistance as has been the case for malaria vectors [27]. Domestic use of insecticide such as aerosols or coils to deter mosquito bites may play a more important role in the selection of insecticide resistance in mosquito populations [37]. However, in Niger and in West Africa generally sources of selection leading to resistant *Ae*. *aegypti* require further investigation.

The allele frequency of 1534C found in Niamey remains lower than elsewhere in West African studies from Ouagadougou [22], Abidjan [24] and Accra [25] where it is at or approaching fixation, similar to Latin American countries such as Colombia [35] and Mexico [41]. The 1016I allele frequency was also much lower in this study than in Angola [20], Abidjan [24], Accra [25], Ouagadougou [22] and Mexico [41]. Similarly, the frequency of 410L was lower than found in recent studies from Ouagadougou [22] and Abidjan [24], though frequencies in Accra [25] and Colombia [35] remain relatively low. In contrast, highest frequencies were reported from Angola [20] and Mexico [42]. Alone, or in combination with F1534C and V1016I, the V410L mutation can confer resistance to pyrethroids [42]. The V1016I and V410L mutations appear linked in *Aedes* populations from Niger. Similar trend has been found in Latin America and in Burkina Faso [22,42]. However, in Ghana [25] the dissociation appears much greater suggesting a different stage of co-evolution for the two mutants [42].

The triple homozygote resistant CIL/CIL was found to be associated with both permethrin and deltamethrin resistance. Similar findings have been reported from Mexico [42], whereas in Ghana this tri-locus genotype has been only associated with permethrin resistance [25]. In this study, the double-locus heterozygote genotype CVV/CIL was found to be associated with both permethrin and deltamethrin resistance, whereas it was associated only with deltamethrin resistance in Mexico [42]. We also found the CIL haplotype to be associated with both permethrin and deltamethrin resistance. In contrast, in Ouagadougou it was associated only with permethrin resistance [22].

The diversity of tri-locus genotypes (10) and tri-locus haplotypes (6) that we found suggest a late emergence of the insecticide resistance compared to Ouagadougou in Burkina Faso where the 1534C mutation is almost fixed, effectively reducing the number of genotypes (6) and haplotypes (3) and to the three mutations [22].

Pre-exposure to the synergist PBO significantly restored susceptibility to both permethrin and deltamethrin insecticides suggesting probable involvement of P450 family genes in the resistance phenotypes [13,37]. Pre-exposure to the synergist DEF also significantly elevated susceptibility to permethrin, though the rise in mortality with deltamethrin exposure was not significant. This is suggestive of the role of esterase family enzymes in pyrethroid resistance [43]. Whilst both target site and metabolic resistance appear to underly pyrethroid resistance in Niamey, metabolic resistance appears to be the main mechanism of resistance in multiple populations from Senegal in which *kdr* mutants were absent [44]. Further studies are required to investigate the spatial extent of resistance/susceptibility profiles across insecticides in Niger, and to determine whether similar resistance mechanisms apply more widely.

## Conclusion

We reported for the first-time multiple insecticide resistance mechanisms including target site mutations and metabolic enzymes among *Ae*. *aegypti* population from Niamey, the largest city of Niger. Our study implicates three target site mutations (F1534C + V1016I + V410L) and metabolic enzymes (oxidases and esterases) as mechanisms involved in resistance to pyrethroids, whilst highlighting susceptibility to alternate insecticide classes. This study provides both important and baseline data for *Ae*. *aegypti* borne disease control and for vector insecticide resistance monitoring respectively in Niamey, Niger.

## Supporting information

S1 Table List of primers sequences used for detecting V410L *kdr* mutation. (DOCX)

S2 Table 1 hour WHO tubes bioassay data. (DOCX)

S3 Table 1 hour bottles bioassay data. (DOCX)

S4 Table Haplotypes and their association with resistance to permethrin of *Ae*. *aegypti*. Significantly associated haplotypes are highlighted in bold.
(DOCX)

S5 Table Haplotypes and their association with resistance to deltamethrin of *Ae*. *aegypti*. Significantly associated haplotypes are highlighted in bold.
(DOCX)

S6 Table Genotypes from AS-PCR and Taqman detection of the kdr mutations. (DOCX)

## References

[1] Agboli E, Zahouli JB, Badolo A, Jöst H. Mosquito-Associated Viruses and Their Related Mosquitoes in West Africa. *Viruses*. 2021; 13 (891). Academic. doi: 10.3390/v13050891 34065928 PMC8151702

[2] Im J, Balasubramanian R, Ouedraogo M, Rosny L, Nana W, Mogeni OD et al. The epidemiology of dengue outbreaks in 2016 and 2017 in Ouagadougou, Burkina Faso. *Heliyon*. Jul. 2020; 6 (7). doi: 10.1016/j.heliyon.2020.e04389 32695907 PMC7364030

[3] Sylla Y, Diane MK, Adjogoua VE, Kadjo H and Dosso M. Dengue Outbreaks in Abidjan: Seroprevalence and Cocirculating of Three Serotypes in 2017. *OSIR Journal*. 2021; 14 (3), pp. 94–10. [Online]. Available: http://www.osirjournal.net/index.php/osir/article/view/233.

[4] Diagne CT, Barry MA, Ba Y, Faye O and Sall AA. Dengue epidemic in Touba, Senegal: Implications for the Grand Magal Pilgrimage for travellers. *J Travel Med*. 2019; 26 (7), pp. 1–2. doi: 10.1093/jtm/tay123 30407572

[5] Amoako N, Duodou S, Dennis FE, Bonney JHK, Asante KP, Ameh J et al. Detection of dengue virus among children with suspected malaria, Accra, Ghana. *Emerg Infect Dis*, Aug. 2018; 24 (8), pp. 1561–1564. doi: 10.3201/eid2408.180341 30015610 PMC6056106

[6] WHO. Yellow Fever—Ghana. Disease outbreaks news. 2021. https://www.who.int/emergencies/disease-outbreak-news/item/yellow-fever—ghana (accessed on august 31^st^ of 2022).

[7] Adogo LY and Ogoh MO. Yellow fever in Nigeria: a review of the current situation. *African Journal of Clinical and experimental Microbiology*. 2020; 21(1), pp. 1–13.

[8] WHO. Disease Outbreak News; Yellow fever in East, West, and Central Africa. 2 september 2022. https://www.who.int/emergencies/disease-outbreak-news/item/2022-DON405 (accessed on february 17^th^ of 2023).

[9] da Silveira LTC, Tura B, and Santos M. Systematic review of dengue vaccine efficacy. *BMC Infect Dis*. 2019; 19 (1), pp. 1–8. 31455279 10.1186/s12879-019-4369-5 PMC6712597

[10] Barrett AD. Yellow Fever in Angola and Beyond—The Problem of Vaccine Supply and Demand. *New England Journal of Medicine*. Jul. 2016; 375 (4), pp. 301–303. doi: 10.1056/NEJMp1606997 27276108

[11] Facchinelli L, Badolo A, and McCall PJ. Biology and Behaviour of *Aedes aegypti* in the Human Environment: Opportunities for Vector Control of Arbovirus Transmission. *Viruses*. Mar. 01, 2023; 15 (3). MDPI. doi: 10.3390/v15030636 36992346 PMC10053764

[12] Badolo A, Sombié A, Yaméogo F, Wangrawa DW, Sanon A, Pignatelli PM et al. First comprehensive analysis of *Aedes aegypti* bionomics during an arbovirus outbreak in west Africa: Dengue in Ouagadougou, Burkina Faso, 2016–2017. *PLoS Negl Trop Dis*. 2022; 16 (7), pp. 1–25. doi: 10.1371/journal.pntd.0010059 35793379 PMC9321428

[13] Badolo A, Sombié A, Pignatelli PM, Sanon A, Yaméogo F, Wangrawa DW et al. Insecticide resistance levels and mechanisms in *Aedes aegypti* populations in and around Ouagadougou, Burkina Faso. *PLoS Neglected Tropical Diseases*. 2019; 13 (5), pp. 1–17. doi: 10.1371/journal.pntd.0007439 31120874 PMC6550433

[14] Messina PJ, Brady OJ, Golding N, Kraemer MUG, Wint GRW, Ray SE et al. The current and future global distribution and population at risk of dengue. *Nat Microbiol*. 2019; 4 (9), pp. 1508–1515. doi: 10.1038/s41564-019-0476-8 31182801 PMC6784886

[15] Egid BR, Coulibaly M, Dadzie SK, Kamgang B, McCall JP, Seda L et al. Review of the ecology and behaviour of *Aedes aegypti* and *Aedes albopictus* in Western Africa and implications for vector control. *Current Research in Parasitology and Vector-Borne Diseases*. Jan. 01, 2022; 2. Elsevier B.V. doi: 10.1016/j.crpvbd.2021.100074 35726222 PMC7612875

[16] Dadzie SK, Akorli J, Coulibaly MB, Alhadji-Dabla KM, Baber I, Bobanga T et al. Building the capacity of West African countries in Aedes surveillance: inaugural meeting of the West African Aedes Surveillance Network (WAASuN). *Parasites&Vectors*. Dec. 2022; 15 (1). doi: 10.1186/s13071-022-05507-0 36271451 PMC9585720

[17] Liu N. Insecticide resistance in mosquitoes: Impact, mechanisms, and research directions. *Annual Review of Entomology*. Jan. 07, 2015; 60. Annual Reviews Inc., pp. 537–559. doi: 10.1146/annurev-ento-010814-020828 25564745

[18] Moyes CL, Vontas J, Martins AJ, Ng LC, Koou SY, Dusfour I et al. Correction: Contemporary status of insecticide resistance in the major Aedes vectors of arboviruses infecting humans. *PLoS Neglected Tropical Diseases*. 2021; 15(1), pp. 1–2. doi: 10.1371/journal.pntd.0009084 33465099 PMC7815121

[19] Haddi K, Tomé HV, Du Y, Valbon WR, Nomura Y et al. Detection of a new pyrethroid resistance mutation (V410L) in the sodium channel of Aedes aegypti: A potential challenge for mosquito control. *Sci Rep*. April, 2017; 7 (46549). doi: 10.1038/srep46549 28422157 PMC5396194

[20] Ayres CF, Seixas G, Borrego S, Marques C, Monteiro I et al. The V410L knockdown resistance mutation occurs in island and continental populations of *Aedes aegypti* in West and Central Africa. *PLoS Negl Trop Dis*. 2020; 14 (5), pp. 1–12. doi: 10.1371/journal.pntd.0008216 32384079 PMC7304628

[21] Fagbohun IK, Oyeniyi TA, Idowu ET, Nwanya O, Okonkwo F, Adesalu KO et al. Detection and Co-occurrence of kdr (F1534C and S989P) Mutations in Multiple Insecticides Resistant *Aedes aegypti* (Diptera: Culicidae) in Nigeria. *J Med Entomol*. 2022; Xx, pp. 1–8. doi: 10.1093/jme/tjac114 35960164

[22] Sombié A, Ouedraogo, Oté M, Saiki E, Sakurai T, Yaméogo F et al. Association of 410L, 1016I and 1534C *kdr* mutations with pyrethroid resistance in *Aedes aegypti* from Ouagadougou, Burkina Faso, and development of a one-step multiplex PCR method for the simultaneous detection of 1534C and 1016I kdr mutations. *Parasites&Vectors*. 2023; pp. 1–10. doi: 10.1186/s13071-023-05743-y 37076920 PMC10116651

[23] Toé HK, Zongo S, Guelbeogo MW, Kamgang B, Viana M, Tapsoba M et al. Multiple insecticide resistance and first evidence of V410L kdr mutation in Aedes (Stegomyia) aegypti (Linnaeus) from Burkina Faso. *Med Vet Entomol*. January 2022; pp. 1–11. doi: 10.1111/mve.12602 35869781

[24] Konan LY, Oumbouke WA, Silué UG, Coulibaly IZ, Ziogba JT, Guessan RKN et al. Insecticide resistance patterns and mechanisms in *Aedes aegypti* (Diptera: Culicidae) populations across Abidjan, Côte d’Ivoire reveal emergent pyrethroid resistance. *J Med Entomol*. 2021; XX, pp. 1–9. doi: 10.1093/jme/tjab045 33876233

[25] Abdulai A, Owusu-Asenso CM, Akosah Brempong, Mohammed AR et al. Insecticide resistance status of *Aedes aegypti* in southern and northern Ghana. *Parasites&Vectors*. Dec. 2023; 16 (1). doi: 10.1186/s13071-023-05752-x 37072865 PMC10111668

[26] Lagare A, Faye M, Fintan G, Fall G, Ousmane H et al. First introduction of dengue virus type 3 in Niger, 2022. *IJID Regions*. Jun. 2023; 7, pp. 230–232. doi: 10.1016/j.ijregi.2023.04.001 37168517 PMC10165393

[27] Czeher C, Labbo R, Arzika I and Duchemin JB. Evidence of increasing Leu-Phe knockdown resistance mutation in Anopheles gambiae from Niger following a nationwide long-lasting insecticide-treated nets implementation. *Malar J*. 2008; 7. doi: 10.1186/1475-2875-7-189 18817574 PMC2562389

[28] PopulationData.net. PopulationData.net. https://www.populationdata.net/pays/niger/airesurbaines (accessed on december 10^th^ of 2020).

[29] WHO. Test procedures for insecticide resistance monitoring in malaria vector mosquitoes. 2016.

[30] Owusu-Asenso CM, Mingle JAA, Weetman D and Afrane YA. Spatiotemporal distribution and insecticide resistance status of *Aedes aegypti* in Ghana. *Parasites&Vectors*. 2022; 15 (1), pp. 1–14. doi: 10.1186/s13071-022-05179-w 35183249 PMC8858493

[31] Abbott W S. A method of computing the effectiveness of an insecticide. *J*. *econ*. *Entomol*. 1925; 18 (2), pp. 265–267.

[32] Brogdon WG and Chan A. Guideline for Evaluating Insecticide Resistance in Vectors Using the CDC Bottle Bioassay. 2012.

[33] WHO. Standard operating procedure for testing insecticide susceptibility of adult mosquitoes in WHO bottle bioassays. January 2022, p. 25 pages.

[34] Sombié A, Saiki E, Yaméogo F, Sakurai T, Shirozu T, Fukumoto S et al. High frequencies of F1534C and V1016I kdr mutations and association with pyrethroid resistance in *Aedes aegypti* from Somgandé (Ouagadougou), Burkina Faso. *Trop Med Health*. 2019; 47 (1), pp. 4–11. doi: 10.1186/s41182-018-0134-5 30787670 PMC6318976

[35] Granada Y, Mejía-Jaramillo AM, Strode C and Triana-Chavez O. A point mutation V419l in the sodium channel gene from natural populations of *Aedes aegypti* is involved in resistance to λ-cyhalothrin in Colombia. *Insects*. 2018; 9 (1). doi: 10.3390/insects9010023 29443870 PMC5872288

[36] Brooks ME, Kristensen K, van Benthem KJ, Magnusson A, Berg CW, Nielsen A et al. glmmTMB Balances Speed and Flexibility Among Packages for Zero-inflated Generalized Linear Mixed Modeling. *The R Journal*. 2017; 9 (2), pp. 378–400.

[37] Amelia-Yap ZH, Sofian-Azirun R, Chen C, Suana I, Lau K, Elia-Amira N et al. Pyrethroids use: Threats on Metabolic-Mediated Resistance Mechanisms in the Primary Dengue Vector *Aedes aegypti* (Diptera: Culicidae). *J Med Entomol*. May 2019; 56 (3), pp. 811–816. doi: 10.1093/jme/tjz007 30715464

[38] Loewenberg S. Niger welcomes largest bednet distribution in history. *The Lancet*. 2006; 367 (9521), p. 1473. doi: 10.1016/S0140-6736(06)68630-3 16683301

[39] Diallo D, Diouf B, Gaye A, Sene NM, Dia I and Diallo M. Dengue vectors in Africa: A review. *Heliyon*. 2022; 8 (5), p. e09459. doi: 10.1016/j.heliyon.2022.e09459 35620619 PMC9126922

[40] Captain-Esoah M, Baidoo PK, Frempong KK, Adabie-Gomez D, Chabi J, Obuobi D et al. Biting behavior and molecular identification of *Aedes aegypti* (Diptera: Culicidae) subspecies in some selected recent yellow fever outbreak communities in Northern Ghana. *J Med Entomol*. Jul. 2020; 57 (4), pp. 1239–1245. doi: 10.1093/jme/tjaa024 32112094

[41] Vera-Maloof FZ, Saavedra-Rodriguez K, Elizondo-Quiroga AE, Lozano-Fuentes S and Black IV WC, “Coevolution of the Ile1,016 and Cys1,534 Mutations in the Voltage Gated Sodium Channel Gene of *Aedes aegypti* in Mexico. *PLoS Negl Trop Dis*. Dec. 2015; 9 (12). doi: 10.1371/journal.pntd.0004263 26658798 PMC4684211

[42] Saavedra-Rodriguez K, Maloof FV, Campbell CL, Garcia-Rejon J, Lenhart A et al. Parallel evolution of vgsc mutations at domains IS6, IIS6 and IIIS6 in pyrethroid resistant *Aedes aegypti* from Mexico. *Sci Rep*. 2018; 8 (1), pp. 1–9. doi: 10.1038/s41598-018-25222-0 29712956 PMC5928250

[43] Schluep SM and Buckner EA. Metabolic resistance in permethrin-resistant Florida *Aedes aegypti* (Diptera: Culicidae). *Insects*. Oct. 2021; 12 (10). doi: 10.3390/insects12100866 34680634 PMC8540271

[44] Sene NM, Marvidis K, Ndiaye EH, Diagne CT, Gaye A, Ngom EHM et al. Insecticide resistance status and mechanisms in *Aedes aegypti* populations from Senegal. *PLoS Negl Trop Dis*. 2021; pp. 1–18, doi: 10.1371/journal.pntd.0009393 33970904 PMC8136859

