# Supplementary material for: First report of V1016I, F1534C and V410L kdr mutations associated with pyrethroid resistance in Aedes aegypti populations from Niamey, Niger
Source: PLoS One. 2024 May 29;19(5):e0304550. doi: 10.1371/journal.pone.0304550 (PMC11135682; doi:10.1371/journal.pone.0304550)
Supplement: S1 Table — (DOCX) [file pone.0304550.s001.docx]

**S1Table: List of primers sequences used for detecting V410L *kdr* mutation**

| Kdr mutation | Primers sequences | References |
| --- | --- | --- |
| V410L | kdr genotyping  PM1_Ext_419F: GAT TCC TCC AGA ACT CCA CC  PM1_Ext_419R: TCA ATG GAT TTG GGT GAC AA  PM1_F_419Wt: CTT GGG TTC GTT CTA CCT TG  PM1_F_419Mut: CTT GGG TTC GTT CTA CCT TT | (Granada et al., 2018) |
