# Supplementary material for: First report of V1016I, F1534C and V410L kdr mutations associated with pyrethroid resistance in Aedes aegypti populations from Niamey, Niger
Source: PLoS One. 2024 May 29;19(5):e0304550. doi: 10.1371/journal.pone.0304550 (PMC11135682; doi:10.1371/journal.pone.0304550)
Supplement: S2 Table — (DOCX) [file pone.0304550.s002.docx]

S2 Table: 1 hour tubes bioassay data

|  |  |  |  |  |  |
| --- | --- | --- | --- | --- | --- |
| **Locality** | | **Insecticide** | **tested** | **dead** | **alive** |
| Niamey | | Permethrin | 96 | 79 | 17 |
| Niamey | | Deltamethrin | 107 | 89 | 18 |
| Niamey | | Bendiocarb | 114 | 114 | 0 |
| Niamey | | Pirimiphos-methyl | 107 | 106 | 1 |
| Niamey | | Malathion | 97 | 97 | 0 |
