# Supplementary material for: First report of V1016I, F1534C and V410L kdr mutations associated with pyrethroid resistance in Aedes aegypti populations from Niamey, Niger
Source: PLoS One. 2024 May 29;19(5):e0304550. doi: 10.1371/journal.pone.0304550 (PMC11135682; doi:10.1371/journal.pone.0304550)
Supplement: S3 Table — (DOCX) [file pone.0304550.s003.docx]

**S6 Table: Genotypes from AS-PCR and Taqman detection of the kdr mutations**

| **Method** | **Insecticide** | **Statut** | **V410L** | **V1016I** | **F1534C** | **Tri-locus** |
| --- | --- | --- | --- | --- | --- | --- |
| Taqman | Control | Alive | VV | VV | FF | VV/VV/FF |
| Taqman | Control | Alive | VL | VI | CC | VL/VI/CC |
| Taqman | Control | Alive | VV | VV | FF | VV/VV/FF |
| Taqman | Control | Alive | VV | VV | FF | VV/VV/FF |
| Taqman | Control | Alive | VV | VV | CC | VV/VV/CC |
| Taqman | Control | Alive | VV | VV | FF | VV/VV/FF |
| Taqman | Control | Alive | VV | VV | FF | VV/VV/FF |
| Taqman | Control | Alive | VV | VV | FC | VV/VV/FC |
| Taqman | Control | Alive | VV | VV | CC | VV/VV/CC |
| Taqman | Control | Alive | VV | VV | FC | VV/VV/FC |
| Taqman | Control | Alive | VL | VI | CC | VL/VI/CC |
| Taqman | Control | Alive | VV | VV | FC | VV/VV/FC |
| Taqman | Control | Alive | VV | VV | FF | VV/VV/FF |
| Taqman | Control | Alive | VL | VI | CC | VL/VI/CC |
| Taqman | Control | Alive | VV | VV | FF | VV/VV/FF |
| Taqman | Control | Alive | VV | VV | FC | VV/VV/FC |
| Taqman | Control | Alive | VV | VV | FF | VV/VV/FF |
| Taqman | Control | Alive | VV | VV | FF | VV/VV/FF |
| Taqman | Control | Alive | VV | VV | FF | VV/VV/FF |
| Taqman | Control | Alive | VV | VV | FC | VV/VV/FC |
| Taqman | Control | Alive | VV | VV | FF | VV/VV/FF |
| Taqman | Control | Alive | VV | VV | FF | VV/VV/FF |
| Taqman | Control | Alive | VV | VV | FC | VV/VV/FC |
| Taqman | Control | Alive | VV | VV | FF | VV/VV/FF |
| Taqman | Control | Alive | VV | VV | FF | VV/VV/FF |
| Taqman | Control | Alive | VV | VV | FC | VV/VV/FC |
| Taqman | Control | Alive | VV | VV | FC | VV/VV/FC |
| Taqman | Control | Alive | VV | VV | CC | VV/VV/CC |
| Taqman | Control | Alive | VV | VV | FF | VV/VV/FF |
| Taqman | Control | Alive | VV | VV | FF | VV/VV/FF |
| Taqman | Control | Alive | VV | VV | FF | VV/VV/FF |
| Taqman | Control | Alive | VV | VV | CC | VV/VV/CC |
| Taqman | Control | Alive | VV | VV | FC | VV/VV/FC |
| Taqman | Control | Alive | VV | VV | FF | VV/VV/FF |
| Taqman | Control | Alive | VV | VV | FF | VV/VV/FF |
| Taqman | Control | Alive | VV | VV | FF | VV/VV/FF |
| Taqman | Control | Alive | VL | VI | FC | VL/VI/FC |
| Taqman | Control | Alive | VV | VV | FC | VV/VV/FC |
| Taqman | Control | Alive | VV | VV | FF | VV/VV/FF |
| Taqman | Control | Alive | VV | VV | FF | VV/VV/FF |
| Taqman | Control | Alive | VV | VV | CC | VV/VV/CC |
| Taqman | Control | Alive | VV | VV | FC | VV/VV/FC |
| Taqman | Control | Alive | VV | VV | FF | VV/VV/FF |
| Taqman | Control | Alive | VV | VV | FF | VV/VV/FF |
| Taqman | Control | Alive | VV | VV | FF | VV/VV/FF |
| Taqman | Control | Alive | VV | VV | FF | VV/VV/FF |
| Taqman | Control | Alive | VV | VV | FC | VV/VV/FC |
| Taqman | Control | Alive | VV | VV | FF | VV/VV/FF |
| Taqman | Control | Alive | VV | VV | FF | VV/VV/FF |
| Taqman | Control | Alive | VV | VV | FC | VV/VV/FC |
| AS-PCR | Perm | Dead | VV | VI | FC | FC/VI/VV |
| AS-PCR | Perm | Dead | VV | VI | FF | FF/VI/VV |
| AS-PCR | Perm | Dead | VV | VI | FF | FF/VI/VV |
| AS-PCR | Perm | Dead | VV | VV | FC | FC/VV/VV |
| AS-PCR | Perm | Dead | VV | VV | FC | FC/VV/VV |
| AS-PCR | Perm | Dead | VV | VV | FC | FC/VV/VV |
| AS-PCR | Perm | Dead | VV | VV | FC | FC/VV/VV |
| AS-PCR | Perm | Dead | VV | VV | FC | FC/VV/VV |
| AS-PCR | Perm | Dead | VV | VV | CC | CC/VV/VV |
| AS-PCR | Perm | Dead | VV | VV | FC | FC/VV/VV |
| AS-PCR | Perm | Dead | VV | VV | FF | FF/VV/VV |
| AS-PCR | Perm | Dead | VV | VV | FC | FC/VV/VV |
| AS-PCR | Perm | Dead | VV | VV | CC | CC/VV/VV |
| AS-PCR | Perm | Dead | VV | VI | FC | FC/VI/VV |
| AS-PCR | Perm | Dead | VV | VV | FC | FC/VV/VV |
| AS-PCR | Perm | Dead | VV | VV | CC | CC/VV/VV |
| AS-PCR | Perm | Dead | VV | VV | CC | CC/VV/VV |
| AS-PCR | Perm | Dead | VV | VV | FF | FF/VV/VV |
| AS-PCR | Perm | Dead | VV | VI | FF | FF/VI/VV |
| AS-PCR | Perm | Dead | VV | VV | FC | FC/VV/VV |
| AS-PCR | Perm | Alive | VV | VI | CC | CC/VI/VV |
| AS-PCR | Perm | Alive | VV | VI | CC | CC/VI/VV |
| AS-PCR | Perm | Dead | VV | VV | FC | FC/VV/VV |
| AS-PCR | Perm | Dead | VV | VV | FC | FC/VV/VV |
| AS-PCR | Perm | Dead | VV | VV | FC | FC/VV/VV |
| AS-PCR | Perm | Dead | VV | VV | FC | FC/VV/VV |
| AS-PCR | Perm | Dead | VV | VV | FF | FF/VV/VV |
| AS-PCR | Perm | Dead | VV | VV | FC | FC/VV/VV |
| AS-PCR | Perm | Dead | VV | VV | FF | FF/VV/VV |
| AS-PCR | Perm | Dead | VV | VV | FF | FF/VV/VV |
| AS-PCR | Perm | Dead | VV | VV | FC | FC/VV/VV |
| AS-PCR | Perm | Dead | VV | VV | FC | FC/VV/VV |
| AS-PCR | Perm | Dead | VL | VI | FC | FC/VI/VL |
| AS-PCR | Perm | Dead | VV | VI | FC | FC/VI/VV |
| AS-PCR | Perm | Dead | VV | VV | FC | FC/VV/VV |
| AS-PCR | Perm | Dead | VL | VI | FC | FC/VI/VL |
| AS-PCR | Perm | Dead | VL | VI | FC | FC/VI/VL |
| AS-PCR | Perm | Dead | VV | VV | FC | FC/VV/VV |
| AS-PCR | Perm | Dead | VV | VI | FC | FC/VI/VV |
| AS-PCR | Perm | Dead | VV | VV | FC | FC/VV/VV |
| AS-PCR | Perm | Dead | VV | VI | FF | FF/VI/VV |
| AS-PCR | Perm | Alive | VV | VV | FC | FC/VV/VV |
| AS-PCR | Perm | Alive | VL | VI | CC | CC/VI/VL |
| AS-PCR | Perm | Alive | VL | VI | CC | CC/VI/VL |
| AS-PCR | Perm | Alive | VV | VV | CC | CC/VV/VV |
| AS-PCR | Perm | Alive | VV | VI | FC | FC/VI/VV |
| AS-PCR | Perm | Alive | VV | VV | CC | CC/VV/VV |
| AS-PCR | Perm | Alive | VV | VV | FC | FC/VV/VV |
| AS-PCR | Perm | Alive | VL | VI | CC | CC/VI/VL |
| AS-PCR | Perm | Alive | VV | VV | CC | CC/VV/VV |
| AS-PCR | Delta | Dead | VL | VI | FC | FC/VI/VL |
| AS-PCR | Delta | Dead | VV | VV | FC | FC/VV/VV |
| AS-PCR | Delta | Dead | VV | VV | FC | FC/VV/VV |
| AS-PCR | Delta | Dead | VL | VI | CC | CC/VI/VL |
| AS-PCR | Delta | Dead | VV | VI | FC | FC/VI/VV |
| AS-PCR | Delta | Dead | VV | VV | FC | FC/VV/VV |
| AS-PCR | Delta | Dead | VV | VV | FF | FF/VV/VV |
| AS-PCR | Delta | Dead | VV | VV | FC | FC/VV/VV |
| AS-PCR | Delta | Dead | VL | VI | FC | FC/VI/VL |
| AS-PCR | Delta | Dead | VV | VV | FF | FF/VV/VV |
| AS-PCR | Delta | Dead | VV | VV | FC | FC/VV/VV |
| AS-PCR | Delta | Dead | VV | VV | FC | FC/VV/VV |
| AS-PCR | Delta | Dead | VV | VI | FF | FF/VI/VV |
| AS-PCR | Delta | Dead | VV | VV | CC | CC/VV/VV |
| AS-PCR | Delta | Dead | VV | VV | FC | FC/VV/VV |
| AS-PCR | Delta | Dead | VV | VV | FC | FC/VV/VV |
| AS-PCR | Delta | Dead | VV | VV | FF | FF/VV/VV |
| AS-PCR | Delta | Dead | VV | VV | CC | CC/VV/VV |
| AS-PCR | Delta | Alive | VV | VI | FC | FC/VI/VV |
| AS-PCR | Delta | Alive | VL | VI | FC | FC/VI/VL |
| AS-PCR | Delta | Alive | VL | VI | CC | CC/VI/VL |
| AS-PCR | Delta | Alive | VV | VV | CC | CC/VV/VV |
| AS-PCR | Delta | Alive | VV | VV | CC | CC/VV/VV |
| AS-PCR | Delta | Alive | VL | VI | CC | CC/VI/VL |
| AS-PCR | Delta | Dead | VV | VV | FC | FC/VV/VV |
| AS-PCR | Delta | Dead | VV | VV | FF | FF/VV/VV |
| AS-PCR | Delta | Dead | VV | VV | FC | FC/VV/VV |
| AS-PCR | Delta | Dead | VV | VV | FF | FF/VV/VV |
| AS-PCR | Delta | Dead | VV | VV | FF | FF/VV/VV |
| AS-PCR | Delta | Dead | VV | VV | FC | FC/VV/VV |
| AS-PCR | Delta | Dead | VV | VV | FC | FC/VV/VV |
| AS-PCR | Delta | Dead | VV | VV | FC | FC/VV/VV |
| AS-PCR | Delta | Dead | VV | VV | FC | FC/VV/VV |
| AS-PCR | Delta | Dead | VV | VV | FF | FF/VV/VV |
| AS-PCR | Delta | Dead | VV | VV | FC | FC/VV/VV |
| AS-PCR | Delta | Dead | VV | VV | FC | FC/VV/VV |
| AS-PCR | Delta | Dead | VL | VI | FC | FC/VI/VL |
| AS-PCR | Delta | Dead | VV | VV | FC | FC/VV/VV |
| AS-PCR | Delta | Dead | VV | VV | FF | FF/VV/VV |
| AS-PCR | Delta | Dead | VV | VV | FC | FC/VV/VV |
| AS-PCR | Delta | Dead | VV | VV | FC | FC/VV/VV |
| AS-PCR | Delta | Dead | VV | VV | FF | FF/VV/VV |
| AS-PCR | Delta | Dead | VV | VV | FC | FC/VV/VV |
| AS-PCR | Delta | Dead | VV | VV | FC | FC/VV/VV |
| AS-PCR | Delta | Dead | VV | VV | FC | FC/VV/VV |
| AS-PCR | Delta | Dead | VV | VV | FC | FC/VV/VV |
| AS-PCR | Delta | Alive | VV | VV | CC | CC/VV/VV |
| AS-PCR | Delta | Alive | VV | VV | CC | CC/VV/VV |
| AS-PCR | Delta | Alive | VV | VV | CC | CC/VV/VV |
| AS-PCR | Delta | Alive | VL | VI | CC | CC/VI/VL |
| AS-PCR | Delta | Alive | VV | VV | FC | FC/VV/VV |
| AS-PCR | Delta | Alive | VV | VV | CC | CC/VV/VV |
| AS-PCR | Delta | Alive | VV | VV | CC | CC/VV/VV |
| AS-PCR | Delta | Dead | VV | VV | CC | CC/VV/VV |
| AS-PCR | Delta | Dead | VV | VV | FF | FF/VV/VV |
| AS-PCR | Delta | Dead | VV | VV | FF | FF/VV/VV |
| AS-PCR | Delta | Dead | VV | VV | FF | FF/VV/VV |
| AS-PCR | Delta | Dead | VL | VI | FC | FC/VI/VL |
| AS-PCR | Delta | Dead | VL | VI | CC | CC/VI/VL |
| AS-PCR | Delta | Dead | VV | VV | FF | FF/VV/VV |
| AS-PCR | Delta | Dead | VV | VV | FC | FC/VV/VV |
| AS-PCR | Delta | Dead | VV | VI | FC | FC/VI/VV |
| AS-PCR | Delta | Dead | VV | VV | CC | CC/VV/VV |
| AS-PCR | Delta | Dead | VV | VV | FC | FC/VV/VV |
| AS-PCR | Delta | Dead | VV | VV | FC | FC/VV/VV |
| AS-PCR | Delta | Dead | VV | VV | FF | FF/VV/VV |
| AS-PCR | Delta | Dead | VV | VV | FF | FF/VV/VV |
| AS-PCR | Delta | Dead | VV | VV | FF | FF/VV/VV |
| AS-PCR | Delta | Dead | VV | VV | FC | FC/VV/VV |
| AS-PCR | Delta | Dead | VV | VV | FF | FF/VV/VV |
| AS-PCR | Delta | Dead | VV | VV | FC | FC/VV/VV |
| AS-PCR | Delta | Dead | VV | VV | FC | FC/VV/VV |
| AS-PCR | Delta | Dead | VV | VI | FC | FC/VI/VV |
| AS-PCR | Delta | Dead | VV | VV | FC | FC/VV/VV |
| AS-PCR | Delta | Dead | VV | VV | FF | FF/VV/VV |
| AS-PCR | Delta | Alive | VV | VV | FC | FC/VV/VV |
| AS-PCR | Delta | Alive | VV | VV | CC | CC/VV/VV |
| AS-PCR | Delta | Alive | VV | VI | FC | FC/VI/VV |
| AS-PCR | Delta | Alive | VL | VI | CC | CC/VI/VL |
| AS-PCR | Delta | Dead | VV | VV | FF | FF/VV/VV |
| AS-PCR | Delta | Dead | VV | VI | FC | FC/VI/VV |
| AS-PCR | Delta | Dead | VV | VV | FC | FC/VV/VV |
| AS-PCR | Delta | Dead | VV | VV | FC | FC/VV/VV |
| AS-PCR | Delta | Dead | VV | VV | FC | FC/VV/VV |
| AS-PCR | Delta | Dead | VV | VV | FF | FF/VV/VV |
| AS-PCR | Delta | Dead | VV | VV | FC | FC/VV/VV |
| AS-PCR | Delta | Dead | VV | VI | FC | FC/VI/VV |
| AS-PCR | Delta | Dead | VV | VV | FC | FC/VV/VV |
| AS-PCR | Delta | Dead | VV | VV | FC | FC/VV/VV |
| AS-PCR | Delta | Dead | VV | VV | CC | CC/VV/VV |
| AS-PCR | Delta | Dead | VV | VV | FC | FC/VV/VV |
| AS-PCR | Delta | Dead | VV | VV | CC | CC/VV/VV |
| AS-PCR | Delta | Dead | VV | VV | FF | FF/VV/VV |
| AS-PCR | Delta | Dead | VV | VV | CC | CC/VV/VV |
| AS-PCR | Delta | Dead | VV | VV | FC | FC/VV/VV |
| AS-PCR | Delta | Dead | VV | VV | FC | FC/VV/VV |
| AS-PCR | Delta | Dead | VV | VV | FF | FF/VV/VV |
| AS-PCR | Delta | Dead | VV | VV | FC | FC/VV/VV |
| AS-PCR | Delta | Alive | LL | VI | CC | CC/VI/LL |
| AS-PCR | Delta | Alive | VV | VV | FC | FC/VV/VV |
| AS-PCR | Delta | Alive | VL | VI | CC | CC/VI/VL |
| AS-PCR | Perm | Dead | VV | VV | FF | FF/VV/VV |
| AS-PCR | Perm | Dead | VV | VV | FC | FC/VV/VV |
| AS-PCR | Perm | Dead | VL | VI | FC | FC/VI/VL |
| AS-PCR | Perm | Dead | VV | VV | FC | FC/VV/VV |
| AS-PCR | Perm | Dead | VV | VV | FC | FC/VV/VV |
| AS-PCR | Perm | Dead | VL | VI | FC | FC/VI/VL |
| AS-PCR | Perm | Dead | VV | VV | FC | FC/VV/VV |
| AS-PCR | Perm | Dead | VV | VV | FF | FF/VV/VV |
| AS-PCR | Perm | Dead | VV | VV | FC | FC/VV/VV |
| AS-PCR | Perm | Dead | VV | VV | FC | FC/VV/VV |
| AS-PCR | Perm | Dead | VV | VV | FF | FF/VV/VV |
| AS-PCR | Perm | Dead | VV | VV | FC | FC/VV/VV |
| AS-PCR | Perm | Dead | VV | VV | FC | FC/VV/VV |
| AS-PCR | Perm | Dead | VV | VV | CC | CC/VV/VV |
| AS-PCR | Perm | Dead | VV | VV | FF | FF/VV/VV |
| AS-PCR | Perm | Dead | VL | VI | FC | FC/VI/VL |
| AS-PCR | Perm | Dead | VV | VV | FF | FF/VV/VV |
| AS-PCR | Perm | Dead | VV | VV | FF | FF/VV/VV |
| AS-PCR | Perm | Dead | VV | VV | FC | FC/VV/VV |
| AS-PCR | Perm | Dead | VV | VV | FC | FC/VV/VV |
| AS-PCR | Perm | Alive | VL | VI | CC | CC/VI/VL |
| AS-PCR | Perm | Alive | LL | II | CC | CC/II/LL |
| AS-PCR | Perm | Alive | VV | VI | FC | FC/VI/VV |
| AS-PCR | Perm | Alive | VV | VI | FC | FC/VI/VV |
| AS-PCR | Perm | Alive | VL | VI | CC | CC/VI/VL |
| AS-PCR | Perm | Dead | VV | VV | FC | FC/VV/VV |
| AS-PCR | Perm | Dead | VV | VV | CC | CC/VV/VV |
| AS-PCR | Perm | Dead | VL | VI | FC | FC/VI/VL |
| AS-PCR | Perm | Dead | VV | VV | FC | FC/VV/VV |
| AS-PCR | Perm | Dead | VL | VI | FC | FC/VI/VL |
| AS-PCR | Perm | Dead | VV | VV | FC | FC/VV/VV |
| AS-PCR | Perm | Dead | VL | VI | CC | CC/VI/VL |
| AS-PCR | Perm | Dead | VV | VV | FC | FC/VV/VV |
| AS-PCR | Perm | Dead | VV | VV | FF | FF/VV/VV |
| AS-PCR | Perm | Dead | VV | VV | CC | CC/VV/VV |
| AS-PCR | Perm | Dead | VV | VV | FC | FC/VV/VV |
| AS-PCR | Perm | Dead | VL | VI | FC | FC/VI/VL |
| AS-PCR | Perm | Dead | VL | VI | FC | FC/VI/VL |
| AS-PCR | Perm | Dead | VV | VV | CC | CC/VV/VV |
| AS-PCR | Perm | Dead | VV | VV | CC | CC/VV/VV |
| AS-PCR | Perm | Dead | VV | VV | FC | FC/VV/VV |
| AS-PCR | Perm | Dead | VV | VV | FF | FF/VV/VV |
| AS-PCR | Perm | Dead | VV | VV | FC | FC/VV/VV |
| AS-PCR | Perm | Dead | VL | VI | FC | FC/VI/VL |
| AS-PCR | Perm | Dead | VL | VI | FC | FC/VI/VL |
| AS-PCR | Perm | Dead | VV | VV | FC | FC/VV/VV |
| AS-PCR | Perm | Dead | VV | VV | FC | FC/VV/VV |
| AS-PCR | Perm | Alive | LL | II | CC | CC/II/LL |
| AS-PCR | Perm | Alive | LL | II | CC | CC/II/LL |
| AS-PCR | Perm | Alive | LL | VI | CC | CC/VI/LL |
| AS-PCR | Perm | Alive | LL | II | CC | CC/II/LL |
