# Supplementary material for: First report of V1016I, F1534C and V410L kdr mutations associated with pyrethroid resistance in Aedes aegypti populations from Niamey, Niger
Source: PLoS One. 2024 May 29;19(5):e0304550. doi: 10.1371/journal.pone.0304550 (PMC11135682; doi:10.1371/journal.pone.0304550)
Supplement: S4 Table — Significantly associated haplotypes are highlighted in bold. (DOCX) [file pone.0304550.s004.docx]

**S4 Table**: Haplotypes and their association with resistance to permethrin of *Aedes aegypti*.

| Number | Haplotypes | Phenotypes | | Total | P-value |
| --- | --- | --- | --- | --- | --- |
|  |  | Dead (Susceptible) | Alive (Resistant) |  |  |
| 1 | FVV | 84 | 8 | 92 | reference |
| 2 | FIV | 4 | 0 | 4 | 1 |
| 3 | **CVV** | 54 | 16 | 70 | **0,014** |
| 4 | **CIV** | 6 | 4 | 10 | **0,016** |
| 5 | **CIL** | 11 | 15 | 26 | **< 10^-6^** |
| Total |  | 159 | 43 | 202 |  |

Significantly associated haplotypes are highlighted in bold
