# Supplementary material for: First report of V1016I, F1534C and V410L kdr mutations associated with pyrethroid resistance in Aedes aegypti populations from Niamey, Niger
Source: PLoS One. 2024 May 29;19(5):e0304550. doi: 10.1371/journal.pone.0304550 (PMC11135682; doi:10.1371/journal.pone.0304550)
Supplement: S5 Table — Significantly associated haplotypes are highlighted in bold. (DOCX) [file pone.0304550.s005.docx]

**S5 Table**: Haplotypes and their association with *Aedes aegypti* resistance to deltamethrin.

| Number | Haplotypes | Phenotypes | | Total | P-value |
| --- | --- | --- | --- | --- | --- |
|  |  | Dead (Susceptible) | Alive (Resistant) |  |  |
| 1 | FVV | 94 | 6 | 100 | reference |
| 2 | FIV | 1 | 0 | 1 | 1 |
| 3 | **CVV** | 56 | 24 | 80 | **< 10^-4^** |
| 4 | CIV | 1 | 1 | 2 | 0,13 |
| 5 | **CIL** | 10 | 8 | 18 | **<0,001** |
| 6 | CVL | 1 | 0 | 1 | 1 |
| Total |  | 163 | 39 | 202 |  |

Significantly associated haplotypes are highlighted in bold
