# Supplementary material for: First report of V1016I, F1534C and V410L kdr mutations associated with pyrethroid resistance in Aedes aegypti populations from Niamey, Niger
Source: PLoS One. 2024 May 29;19(5):e0304550. doi: 10.1371/journal.pone.0304550 (PMC11135682; doi:10.1371/journal.pone.0304550)
Supplement: S6 Table — (DOCX) [file pone.0304550.s006.docx]

S3 Table: 1 hour bottles bioassay data

|  |  | |  |  |  |  |
| --- | --- | --- | --- | --- | --- | --- |
| **Locality** | | **Insecticide** | | **tested** | **dead** | **alive** |
| Niamey | | Permethrin | | 105 | 71 | 34 |
| Niamey | | PBO+Permethrin | | 100 | 99 | 1 |
| Niamey | | DEF+ Permethrin | | 111 | 106 | 5 |
| Niamey | | Deltamethrin | | 110 | 84 | 26 |
| Niamey | | PBO+deltamethrin | | 84 | 82 | 2 |
| Niamey | | DEF+Deltamethrin | | 101 | 83 | 18 |
