# Supplementary material for: First report of V1016I, F1534C and V410L kdr mutations associated with pyrethroid resistance in Aedes aegypti populations from Niamey, Niger
Source: PLoS One. 2024 May 29;19(5):e0304550. doi: 10.1371/journal.pone.0304550 (PMC11135682; doi:10.1371/journal.pone.0304550)
Supplement: Attachment — Submitted filename: Response to Reviewers 25.11.2023.docx [file pone.0304550.s007.docx]

**Responses to Reviewers**

Reviewer #1: 1. Introduction: 'Data on Aedes aegypti bionomics and insecticide resistance status are still lacking' - There have been various insecticide resistance reports in Aedes aegypti in West Africa.

Thank you for this suggestion, we agree with the reviewer that there are recent reports on insecticide resistance of Aedes aegypti from West Africa, though these are limited to a few countries, and so data on Aedes aegypti bionomics are still lacking. We rephrased the sentence accordingly to give a more balanced representation as suggested. (Line 76-78)

2. Methods: Bioassay tests were performed according to World Health Organizations (WHO) protocol using Anopheles diagnostic doses [27] - Justify why Anopheles diagnostic doses were used when Aedes diagnostic doses are available for resistance testing.

We have added the following sentence to explain this and clarify interpretation ‘Due to the absence of WHO-recommended doses for Ae. aegypti mosquitoes at the time of bioassays, Anopheles diagnostic doses were used (WHO 2016). Though these doses are slightly higher than Aedes doses and so provide a conservative assessment of resistance, they have been commonly used (Moyes et al, 2017; Badolo et al, 2019; Owusu-Asenso et al, 2022, Abdulai et al, 2023)‘ (Line 127-130)

3. Methods: Temperature and humidity during the tests were not stated.

Temperature and humidity conditions during tests were mentioned in mosquito rearing section (Line 123-124). We have now repeated these conditions in the bioassay section for more clarity (Line 140, 161-163).

4. Methods: How many samples were used for Allele-specific - PCR for the detection of F1534C, V1016I and V410L mutation

A total of 202 mosquitoes, including both survivors and dead were selected from exposure to 0.75% permethrin and 0.05% deltamethrin bioassays for F1534C, V1016I and V410L kdr mutations detection; this number is specified in the ‘Allele-specific - PCR for the detection of F1534C, V1016I and V410L mutations’ section of the Methods. (Line 177-180)

5. Methods: What were the modifications done on the protocol of Granada et al.  2018?

The Modifications on the protocol of Granada et al, 2018 were related to the PCR conditions as mentioned in Sombié et al, (2023). Conditions are given in the ‘Allele-specific - PCR for the detection of F1534C, V1016I and V410L mutations’ section of the Methods. (Line 180-188)

6. Methods, Results, Discussion: 'Elevated activity of oxidase and esterase enzymes were detected and may also be involved in metabolic resistance to pyrethroids' was mentioned in the abstract but the methodologies and results were not mentioned and discussed. The elevation of activity should not be based on a synergist test.

Yes, it is true that synergist test allows to test the implication or not of metabolic enzymes in the resistance but can’t allow to quantify the level of these enzymes. Sentences related to the involvement of synergists in the results and discussion now indicate possible involvement of enzymes rather than elevated activity. (Line 51-53, 287-293, 351-356)

7. Results: 'A total of 10 tri-locus combinations genotypes were found out of 27 possible (Figure 4).' - Rewrite to clarify.

The sentence is rephrased, as ‘Ten genotypes were found out of 27 possible (Figure 4).genotype combinations across the three kdr mutations F1534C, V1016I and V410L (Fig 3).’(Line 227-229)

8. What was the history of insecticide use in the study areas?

Ae aegypti has not been specifically targeted by any vector control intervention, as is the case in many countries of West Africa. However, we now include a paragraph (2nd para of Discussion) to discuss possible sources whilst indicating that uncertainty remains and further research is required (321-326)

Reviewer #2:

1. Please add numbered lines throughout the manuscript; pointing out the reviewer's comments is difficult.

The manuscript now has numbered lines.

2. Methodology
CDC bottle bioassays interpretations
It would be necessary to update the interpretation of the resistance status based on the % mortality in the bottle bioassays. Since the authors refer to the WHO 2016 manual for selecting DD, in the same manual, the interpretation of the results in bottle and cylinder of the WHO is established in the same way.

We rephrased this sentence according to the reviewer’s suggestion and now omit the term CDC from the bottle bioassays since these are following WHO methods and interpretations. (Lines 203-207)

The methodology for detecting kdr mutations is confusing. Please clarify. AS-PCR should be detailed. Please describe the primers used. Are you comparing two methods?

To avoid long explanation in the methodology, we refered to papers published where the same method is used. However, we tried to develop a bit more for clarification.

The objective was not to compared AS-PCR vs qPCR methods, we have used qPCR method on F0 populations to give an idea of the kdr mutations frequencies in the original population. The difference between the two sets of results in terms of allele frequencies (added to Table 1) is likely due to the different cohorts of mosquitoes used, but both indicated that all resistant alleles were present'. We have added this clarification to the Results sub-section Alleles and genotypes of kdr mutations and their association with pyrethroid resistance (Lines 225-228)

Which mosquitoes were submitted to each method? You said that 50 non-exposed mosquitoes were genotyped by qPCR as described in the methodology but in AS-PCR, what type of mosquitoes were used, and how many?

A total of 202 mosquitoes including alive and dead from permethrin 0.75% and deltamethrin 0.05% exposure were selected for AS-PCR genotyping to study the relationship between kdr mutations and survivorship. 50 mosquitoes not exposed to insecticides were used for genotyping these mutations by using TaqMan qPCR. This is added to Table 1 and in the text (Lines 177-179) and (Lines 190-191).

What do you mean by exposed mosquitoes, alive or dead?

Mosquitoes are exposed to insecticide, 25/tube and the number or dead and alive mosquitoes are recorded at the end of bioassay 24h later. We took all dead and alive mosquitoes from 2 tubes of deltamethrin and permethrin for the molecular analysis.

What do you mean by non-exposed mosquitoes, field mosquitoes?

Please explain it the detailed in the methodology.

All the mosquitoes used were F0 or F1 or F2 mosquitoes from respectively field-collected-eggs hatching and offspring of F0. F0 non exposed to insecticide mosquitoes (50) were used to estimate the distribution of the kdr mutation in the population. We clarified that in the methodology, in the subsection ‘TaqMan detection of F1534C, V1016I and V410L kdr mutations:’(Lines 190-191)

3. Results
It is confusing the way of expressing the kdr genotypes. Please use two alleles for each locus.

We made necessary changes with Kdr genotypes expressed using the two alleles of each locus, only haplotypes are expressed differently.

The tri-locus haplotypes CIL, CVV, and CIV were significantly associated with permethrin resistance ( p < 10-6 , p = 0.014 and p = 0.016, respectively) (S3 table).
Those results are in Table 2.

The results presented in the table 2 are related to the genotypes while the data of S3 table are related to the haplotypes association with phenotypic resistance.

Discussions are limited to investigations done on the continent. There is a vast literature on the association of kdr mutations and pyrethroid resistance worldwide that should be included.

We mainly focus the discussion in Africa where dengue is a growing concern without enough evidence du support its control. However, we included some references now for Aedes kdr-based resistance to insecticide outside Africa. (Lines 337-348)

**Reviewer #3:**

1. The authors state bioassays were conducted as per Ref #29 - Guideline for Evaluating Insecticide Resistance in Vectors Using the CDC Bottle Bioassay. While the stated diagnostic was used for the CDC bottle bioassays, the authors state that mosquitoes were exposed to the insecticides for 1 hour while the diagnostic timepoint for the CDC bottle bioassays at the diagnostic concentration used is stated as 30 minutes in the reference. The authors do not explain why this modification was made in the submitted manuscript.

The objective of the bottles bioassay here was to assess how pre exposure to synergist affect pyrethroid efficacy. The methodology follows the WHO version of the bottle bioassays and so to avoid confusion we have removed reference to them as CDC bottle bioassays Lines (143-145) and (Lines 203-207)

2. Throughout the manuscript - the authors alternate between "Ae aegypti" and "Aedes aegypti" - please consider improving consistency

The correction is made throughout the document.

3. Knockdown should be defined.

Knock down is defined in the Introduction (Lines 80-82)

4. Clarification is required on whether knockdown was recorded for a total of 60 minutes throughout the exposure period or if knockdown was recorded after the 60 min exposure.

Knockdown was recorded every 10 minutes during the 60 minutes exposure period but data for this are not presented and to avoid confusion we do not make reference to this. Mortality was recorded after 24h, and this is the endpoint we use throughout in line with the WHO version of the bottle bioassay protocol.

5- Allele specific PCR - please specify what modifications were made to the published protocol.

We have addressed this above

6- Statistical analysis - "Mortalities" should be "mortalities"

The correction has been made. We have also corrected interpretation of the synergist bioassays in this section to follow WHO recommendations, but this does not affect any interpretations of the results (Lines 203-207)

7. Major consistency and formatting errors in the references section.

The references have been revised and corrected.
